# Biologically defined neuronal synuclein disease as a tool to advance drug development

**DOI:** 10.1038/s41531-024-00845-5

**Published:** 2024-12-20

**Authors:** Gennaro Pagano, Tien Dam, Geoffrey A. Kerchner, Wendy R. Galpern, Milton Biagioni, Rajesh Karan, Danna Jennings, M. Judith Peterschmitt, Tania Nikolcheva, Patrik Brundin

**Affiliations:** 1 Roche Pharma Research and Early Development (pRED), Roche Innovation Center, F. Hoffmann-La Roche Ltd., Basel, Switzerland; 2 University of Exeter Medical School, London, UK; 3 Neumora Therapeutics, Watertown, MA USA; 4 Janssen Research & Development, LLC, Cambridge, MA USA; 5 UCB Pharma, Brussels, Belgium; 6 Global Drug Development, Novartis Pharma AG, Basel, Switzerland; 7 Denali Therapeutics Inc., South San Francisco, CA USA; 8 Sanofi, Clinical Development Neurology, Cambridge, MA USA; 9 Product Development Neurology, F. Hoffmann-La Roche Ltd., Basel, Switzerland

**Keywords:** Biomarkers, Neurodegenerative diseases, Drug discovery, Biomarkers

## Abstract

In a recent Viewpoint article (*JAMA Neurol*. 2024;81:789‒90), Okubadejo et al. raised concerns regarding two recent proposals for biological definitions and staging systems for synucleinopathies (the Neuronal Synuclein Disease Integrated Staging System and SynNeurGe system). While acknowledging these concerns, we provide an alternative perspective—that such frameworks represent important steps forward by allowing biologically defined populations to be targeted with precision treatments that can be accurately evaluated using stage-specific outcomes.

Okubadejo, Okun, and Jankovic^1^ raise legitimate concerns regarding the two recent proposals for biological definitions and staging systems for synucleinopathies, namely the Neuronal Synuclein Disease Integrated Staging System (NSD-ISS)^2^ and the SynNeurGe system^3^.

The authors emphasize several important challenges: (1) the biomarkers used to biologically define the populations are currently not widely accessible or affordable, especially in underserved communities; (2) in the current form, these biomarkers require invasive sampling (cerebrospinal fluid [CSF] for the alpha-synuclein seed amplification assay [SAA]); (3) the resulting readouts are binary and not quantitative; (4) the biomarkers do not reflect the type or severity of symptoms; and (5) asymptomatic, but biomarker positive, individuals are included in the disease classification.

Despite the above caveats, there are valuable applications for these frameworks which warrant consideration: while the novel biological definitions can be expected initially to be used in a clinical research context, they could become more broadly utilized by the development of more accessible approaches to identifying pathological alpha-synuclein, e.g., skin biopsy or blood markers^4^. In addition, there are currently great inequities for those suffering from synucleinopathies between and within countries^5^ because only a minority has access to specialists who can accurately diagnose Parkinson’s disease (PD) and dementia with Lewy bodies (DLB)^6,7^. The development of a validated biomarker for neuronal synucleinopathy in an easily accessible fluid, such as blood, could potentially facilitate diagnosis, including for those without access to clinical specialists.

In addition, we believe staging systems are powerful tools that will *accelerate drug development* in neuronal synucleinopathy by allowing clinical trials that enroll *biologically defined populations* to be conducted and by enabling the use of *stage-dependent outcome measures*. During the past decade, clinicians and scientists have emphasized the heterogeneity of PD, regarding both symptomatology and underlying molecular pathogenesis, and the similarities between PD dementia (PDD) and DLB have been stressed^8^^,9,10^. This heterogeneity may have contributed to the failure of several trials of experimental neuronal synuclein-targeted therapies aimed at slowing the progression of symptoms. Consequently, many investigators have called for a precision medicine approach when developing new drugs for PD, PDD, and DLB, and concerns regarding evaluating drugs in populations defined only by clinical symptomatology have been raised^2,3,11,12^. *Instead, the solution could be to target the right population (biologically defined) with the right drugs (that match that biology) and to evaluate the right outcomes (stage-specific) that change sufficiently within the duration of the clinical trial to allow detection of a potential slowing of* *progression*^2,3,12^. For example, implementing NSD-ISS could be a first step toward increasing the probability of success of future clinical trials in neuronal synucleinopathy. An ideal population to test a drug designed to slow or even stop disease progression is biologically defined by the presence of pathological alpha-synuclein and only early signs of dopaminergic neurodegeneration, with minimal signs or symptoms of disease, and no functional impairment, representing Stage 2 A and 2B of NSD-ISS^2^. As SAA becomes more widely available at research sites, the NSD-ISS framework can be expected to facilitate trials of drugs in the earliest stages of the disease, prior to significant neurodegeneration, which may increase the magnitude and the impact of a therapeutic effect.

The paradigm shift of the two new biological frameworks is that the *disease is defined by biology and not by symptomatology*. This approach will reduce the heterogeneity of the trial participants in terms of underlying molecular pathogenesis and more likely achieve slowing of disease progression by targeting the underlying biology of the disease. Notwithstanding these strengths, there are still uncertainties on how long it would take for a population to progress from one stage to the next, e.g., from NSD-ISS stage 2B (very early stage before the onset of functional deficits) to Stage 3 (functional motor or cognitive deficits).

The NSD-ISS framework is an excellent starting point for a biological definition of neuronal synucleinopathy and can be applied to clinical trials now. Increasing the power of clinical trials by reducing the heterogeneity of the population and focusing on the right biology and the right endpoints will advance the field. We must urgently address the unmet need for early-stage treatments that slow disease progression in neuronal synucleinopathies, which are clinically diagnosed today as PD, PDD, DLB, or prodromal PD (asymptomatic SAA+). A clear advantage of using the NSD-ISS is that it allows the testing of drugs in neuronal synucleinopathy broadly, regardless of whether motor or cognitive symptoms present first. NSD-ISS may also increase awareness of early PD signs and symptoms (e.g., hyposmia, sleep disturbance), and bring individuals with these findings to medical attention and clinical trials sooner. Similar personalized medicine approaches are already underway in genetically defined PD, e.g., *LRRK2* inhibitors are tested in individuals with *LRRK2* mutations^13^, and experimental treatments that enhance glucocerebrosidase activity are tested in people with genetic risk variants in the *GBA1* gene^14^. Important lessons can be learned from the development of biomarkers for Alzheimer’s disease. The first amyloid positron emission tomography (PET) imaging tool was developed two decades ago and was initially used to reduce heterogeneity in clinical trials^15^. Eventually, amyloid PET tracers achieved FDA approval for Alzheimer’s diagnosis, as did novel CSF-based biomarkers of amyloid neuropathology, and blood-based biomarkers are now following suit^16^. Today, the presence of amyloid positivity is considered a prerequisite for accessing newly approved therapies targeting amyloid^17^. These developments in the Alzheimer’s field demonstrate that the availability of biomarkers for diagnosis in everyday clinical practice should be considered a consequence of—not a prerequisite for—their use in research studies aimed at developing new therapies. The FDA recently issued a Letter of Support regarding the use of CSF SAA as an enrichment biomarker, noting the potential impact of enrolling a biologically defined population in trials evaluating compounds targeting synuclein^18^.

We believe that continuing these first steps into a modern era of precision medicine for neurodegenerative diseases should be encouraged. We agree that equity in healthcare access is of paramount importance, but ultimately the new scientific developments are likely to lead to better and targeted treatments, with improved quality of life for greater numbers of patients. Fewer patients will be left out as testing becomes more accessible and targeted therapies are developed. We acknowledge that in order to impact the lives of greater numbers of patients and not further widen existing healthcare disparities, biomarker accessibility issues need to be considered early on. Thus, ultimately, the research and industry communities must develop validated tests that are both affordable and can be applied in readily accessible biomatrices (e.g., blood and saliva). While such assays might take several years to develop and fully validate, this should not hinder the use of existing assays already today in research and clinical trial settings. For all these reasons, with the recent advances in biomarkers for alpha-synuclein aggregates, we suggest that it is time to accelerate the adoption of a biological definition of neuronal synucleinopathy, rather than tap on the brakes. While symptom-based classifications and assessments will remain valuable when managing patients’ symptoms, slowing or ignoring these innovations regarding biomarkers will certainly delay or hinder innovative and transformative medicines reaching patients in need.

In summary, today the NSD-ISS is a valuable clinical research tool when developing new drugs for synucleinopathies. Although more needs to be done, including working with all relevant stakeholders, this framework holds promise as a clinical practice tool that can help identify individuals earlier in the course of the neurodegenerative process who may be more likely to respond to novel therapies that slow disease progression, ultimately allowing for potential additional benefits of early intervention.

## Data Availability

No datasets were generated or analyzed during the current study.
